# The value of QT interval in differentiating vasovagal syncope from epilepsy in children

**DOI:** 10.1186/s13052-022-01388-2

**Published:** 2022-12-12

**Authors:** Xin Wang, Shuo Wang, Haihui Xiao, Runmei Zou, Hong Cai, Liqun Liu, Fang Li, Yuwen Wang, Yi Xu, Cheng Wang

**Affiliations:** 1 Department of Pediatric Cardiovasology, Children’s Medical Center , The Second Xiangya Hospital, Central South University, No.139 Renmin Middle Road, Hunan 410011 Changsha, China; 2 Department of Pediatrics , The Eighth Affiliated Hospital, Sun Yat-sen University, Fu tian, Guangdong 518033 Shenzhen, China; 3 Department of Neonatology , Xiangya Hospital, Central South University, Hunan 410008 Changsha, China; 4 Department of Pediatrics, Loudi Central Hospital, 417099 Loudi, China; 5 Department of Pediatric Neurology, Children’s Medical Center, The Second Xiangya Hospital, Central South University, 410011 Changsha, China

**Keywords:** Vasovagal syncope, Epilepsy, Electrocardiography, QT interval, Differential diagnosis, Children

## Abstract

**Background:**

Both vasovagal syncope (VVS) and epilepsy present with transient loss of consciousness and are often difficult to identify. Hence this study aimed to explore the value of QT interval in the differentiation of VVS and epilepsy in children.

**Methods:**

One hundred thirteen children with unexplained transient loss of consciousness were selected. 56 children with VVS (VVS group), including 37 males and 19 females, the average age is 9.88 ± 2.55 years old. 57 children with epilepsy (epilepsy group), including 36 males and 21 females, the average age is 8.96 ± 2.67 years old. At the same time, the 60 healthy individuals (control group) were examined according to age and sex. The QT interval of 12-lead electrocardiogram in a basal state of three groups was measured and statistically analyzed by SPSS 24.0 software.

**Results:**

Compared with the control group, (1) QTcmax, QTcmin and QTcd were significantly longer in VVS group (*P* < 0.05), QTmax and QTmin were significantly shorter in VVS group (*P* < 0.05), and there were no significant differences in QTd between the two groups (*P* > 0.05). (2) The QTmax and QTmin were significantly shorter in epilepsy group (*P* < 0.05), and there were no significant differences in QTd, QTcmax, QTcmin, QTcd between the two groups (*P* > 0.05). Compared with the epilepsy group, The QTcmax, QTcmin, QTcd were significantly longer in VVS group (*P* < 0.05), and there were no significant differences in QTd, QTmax, QTmin between the two groups (*P* > 0.05). When QTcmax > 479.84 ms, QTcmin > 398.90 ms and QTcd > 53.56 ms, the sensitivity and specificity of diagnosing VVS were 62.5% and 77.19%, 82.14% and 50.88%, 82.14% and 38.60% respectively.

**Conclusion:**

QTcmax, QTcmin and QTcd have certain value in differentiating VVS from epilepsy in children.

**Supplementary Information:**

The online version contains supplementary material available at 10.1186/s13052-022-01388-2.

## Background

Transient loss of consciousness (TLOC) is a spontaneous and transient loss of consciousness accompanied by a rapid and complete recovery [1, 2]. TLOC is common in children, with 15% of them experiencing TLOC at least once before the age of 18 [2, 3]. Syncope and epilepsy are the main causes of TLOC in children [2].

Syncope is due to transient global cerebral hypoperfusion caused by a variety of reasons, it is characterized by a TLOC, the decrease in body muscle strength cannot maintain the autonomous posture to fall [1, 4], and is a common emergency in children, accounting for 1%~2% of emergency departments visits [1]. Neurally mediated syncope (NMS) is the most common form in children, accounting for about 70%~80% [5]. Vasovagal syncope (VVS) is the most common in NMS [6], which is divided into three hemodynamic types, including vasoinhibitory type vasovagal syncope (VVS-VI), cardioinhibitory type vasovagal syncope (VVS-CI) and mixed type vasovagal syncope (VVS- M). Epilepsy is a group of chronic brain diseases characterized by transient dysfunction of the central nervous system caused by abnormal discharges of neurons in the brain, with the characteristics of sudden occurrence and repeated seizures [7]. Syncope and epilepsy are both common pediatric emergencies, their pathogenesis and treatment principles are different, but both of them can be characterized as TLOC, which is difficult to distinguish [7]. Syncope and epilepsy are identified clinically by detailed medical history, physical examination, electrocardiogram (ECG), head-up tilt test (HUTT), electroencephalogram (EEG) and cranial MRI. However, the recurrence of syncope in HUTT has the risks of temporary aphasia [8], convulsion [9], arrhythmia [10], psychological fear [11] and so forth. Therefore it is very important to find simple methods and easily available markers to assist in differential diagnosis.

Electrocardiogram can reflect the changes of autonomic nervous function and is a popular examination technique in clinic. QT interval is the total duration of cardiomyocyte depolarization and repolarization, which is regulated by autonomic nerve. QT interval dispersion (QTd) can reflect the inconsistency of ventricular repolarization and is of great significance in predicting the occurrence of malignant arrhythmias [12]. QT interval is simple and easy to obtain in clinic. This paper studies the difference of QT interval index between VVS and epilepsy in children, and discusses the value of QT interval index in the differentiation of VVS and epilepsy in such population.

## Methods

### Participants

One hundred thirteen children with unexplained TLOC in the Department of Pediatric Cardiology and Neurology, The Second Xiangya Hospital, Central South University, from May 2018 to December 2020 were selected through detailed history collection, physical examination, blood examination, immune and metabolic disease screening, 12-lead ECG and 24-hour ambulatory electrocardiogram (A-ECG), echocardiogram, electroencephalogram (EEG), head MRI/CT, HUTT, excluding patients with psychiatric, metabolic, liver, kidney, immune diseases, psychogenic diseases, history of heart and lung disease. There were 56 children with VVS (VVS group), 37 males and 19 females, average age of 9.88 ± 2.55 years old, and 57 children with epilepsy (epilepsy group), 36 males and 21 females, average age of 8.96 ± 2.67 years old. In the same period, 60 healthy individuals (control group) were examined according to age and sex, including 40 males and 20 females, average age of 9.43 ± 2.36 years old. The Medical Ethical Committee at The Second Xiangya Hospital, Central South University, approved this research. We obtained informed consent from the guardians of the children.

### Electrocardiogram

Before examination, the cardiovascular active drugs were discontinued for 5 half-lives, and the drugs that affected the autonomic nervous function were discontinued. The children lay flat on the examination bed to rest above 10 min, and the synchronous 12-lead body surface ECG was recorded with ECG monitoring software system from Beijing Standley Technology Co, Ltd (Beijing, China). ECG gain 1 mV = 10 mm, paper speed 25 mm/s.

### Measurement of QT interval index

QT interval: ECG professionals use computer automatic measurement combined with manual intervention to select the ECG with clear waveform. Taking the starting level of Q wave as the equipotential line, the time from the start point of QRS wave to the end point of T wave is measured, and the maximum QT interval (QTmax) and minimum QT interval (QTmin) of 12 leads were measured. The method of judging the end point of T wave is as follows: the intersection of T wave and equipotential line; if there is U wave, it is the notch between T wave and U wave; the biphasic T wave finally returns to the intersection of equipotential line [12]. Each index was measured continuously for three cardiac cycles and the average value was taken. Corrected QT interval (QTc): QTc has been obtained by Bazett’s Square Root Correction Formula (QTc = QT/RR^0.5). QTd: It is the difference between QTmax and QTmin in 12-lead ECG, namely QTd = QTmax-QTmin. Corrected QT interval dispersion (QTcd): It is the difference between QTcmax and QTcmin in 12-lead ECG, namely QTcd = QTcmax- QTcmin.

### Statistical method

Statistical analysis was performed using the Statistical Package for Social Sciences (SPSS) version 24.0 software for Windows (IBM Corp, Armonk, New York). The measurement date of normal distribution was expressed by mean ± SD. One-way analysis of variance (ANOVA) was used for comparison among the three groups, and LSD-t test was used for pairwise comparison. The counting data were expressed by the number of cases and percentage (%), and the comparison between groups is expressed by the *χ*^2^ test. The receiver operating characteristic (ROC) curve was drawn to evaluate the efficacy of QT interval in the differential diagnosis of VVS and epilepsy in children. Z test was used to compare the area under the curve (AUC). *P* < 0.05 was considered statistically significant.

## Results

### General data comparison

**Fig. 1:**
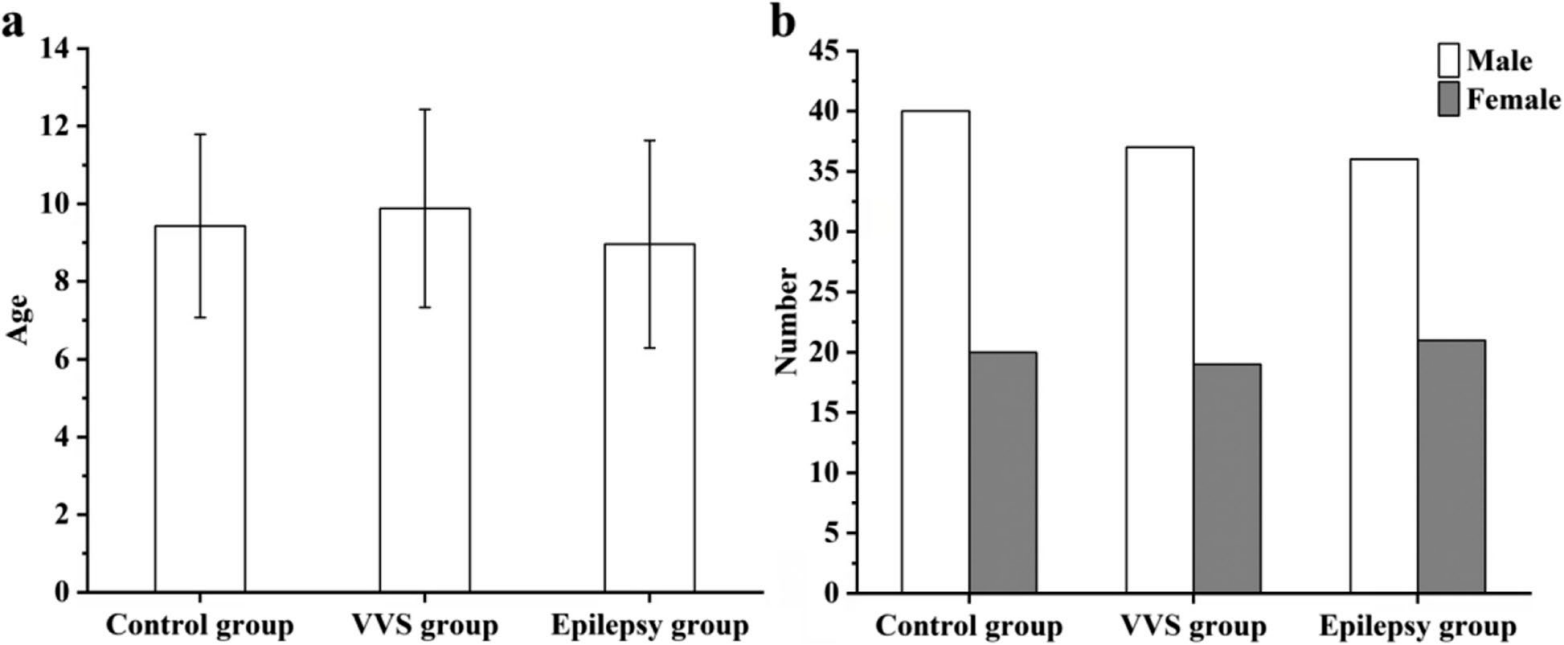
Comparison of age and sex in VVS group, epilepsy group and control group. Note: **a**: age comparison. **b**: sex comparison

One hundred seventy three children were included in this study. There were 56 patients in VVS group (29 patients in VVS-VI, 9 patients in VVS-CI, 18 patients in VVS-M), including 37 males and 19 females, the age was 4 ~ 14 years old, and the average age was (9.88 ± 2.55) years old. There were 57 patients in epilepsy group, including 36 males and 21 females, the age was 3 ~ 14 years old, and the average age was (8.96 ± 2.67) years old. There were 60 healthy individuals in the control group, including 40 males and 20 females, the age was 3 ~ 14 years old, and the average age was (9.43 ± 2.36) years old. There were no significant differences among the three groups in age (*F* = 1.821, *P* = 0.165) and sex (*χ*^2^ = 0.180, *P* = 0.914), as shown in Fig. 1.

### Comparison of QT interval index between groups

**Fig. 2:**
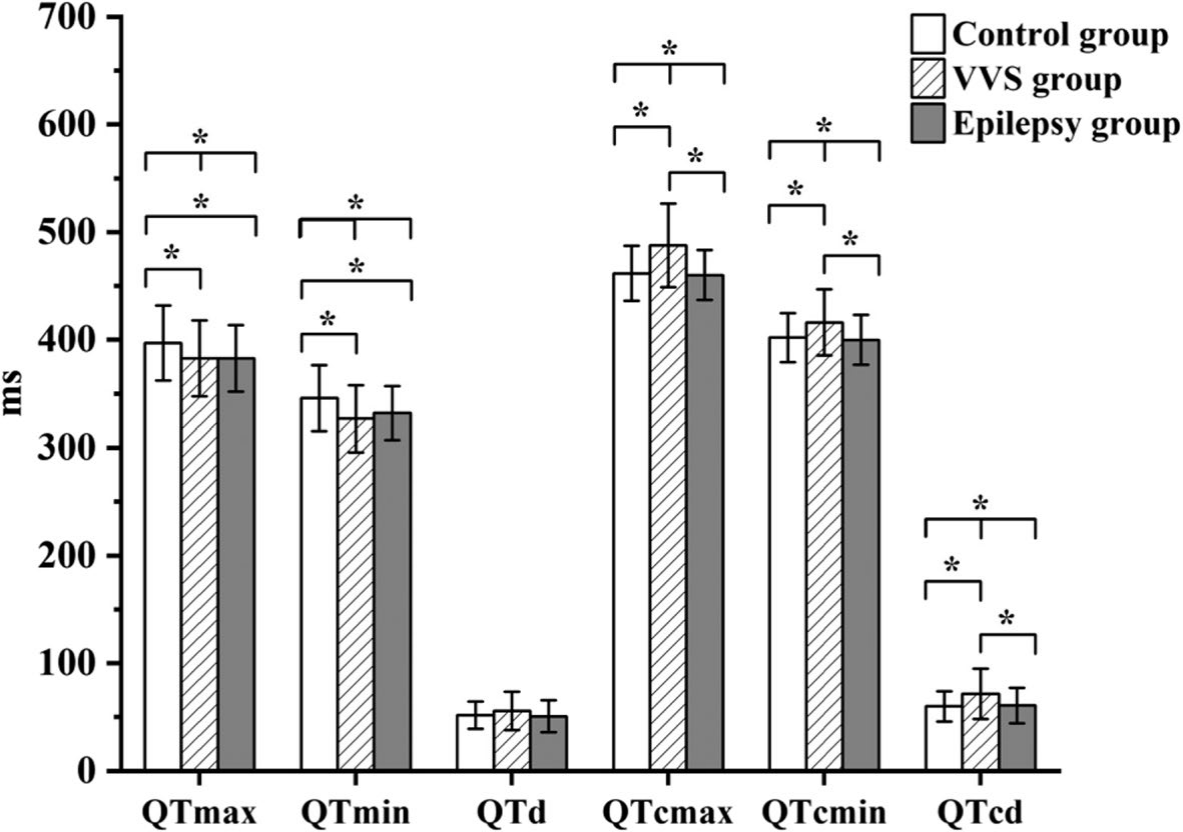
Comparison of QT interval indexes in VVS group, epilepsy group and control group. Note: VVS: Vasovagal syncope. QTmax: maximum QT interval. QTmin: minimum QT interval. QTd: QT interval dispersion. QTcmax: corrected maximum QT interval. QTcmin: corrected minimum QT interval. QTcd: corrected QT interval dispersion. *: *P* < 0.05

There were significant differences in QTmax, QTmin, QTcmax, QTcmin and QTcd among the three groups (*P* < 0.05), but there was no significant difference in QTd among the three groups (*P* > 0.05). Compared with the control group, QTcmax (*P* = 0.000), QTcmin (*P* = 0.004) and QTcd (*P* = 0.001) were significantly prolonged and QTmax (*P* = 0.022) and QTmin (*P* = 0.001) were significantly shortened in VVS group (*P <* 0.05), there was no significant difference in QTd (*P* = 0.130) between the VVS and control groups (*P >* 0.05). QTmax (*P* = 0.023) and QTmin (*P* = 0.012) in epilepsy group were significantly shorter than those in control group (*P <* 0.05), and there was no significant difference in QTd (*P* = 0.734), QTcmax (*P* = 0.981), QTcmin (*P* = 0.659) and QTcd (*P* = 0.833) between the epilepsy and control groups (*P* > 0.05). QTcmax (*P* = 0.000), QTcmin (*P* = 0.001) and QTcd (*P* = 0.002) in VVS group were significantly longer than those in epilepsy group (*P* < 0.05). There was no significant difference in QTd (*P* = 0.068), QTmax (*P* = 0.983) and QTmin (*P* = 0.324) between the VVS and epilepsy groups (*P* > 0.05), as shown in Fig. 2.

### ROC curve analysis

**Fig. 3:**
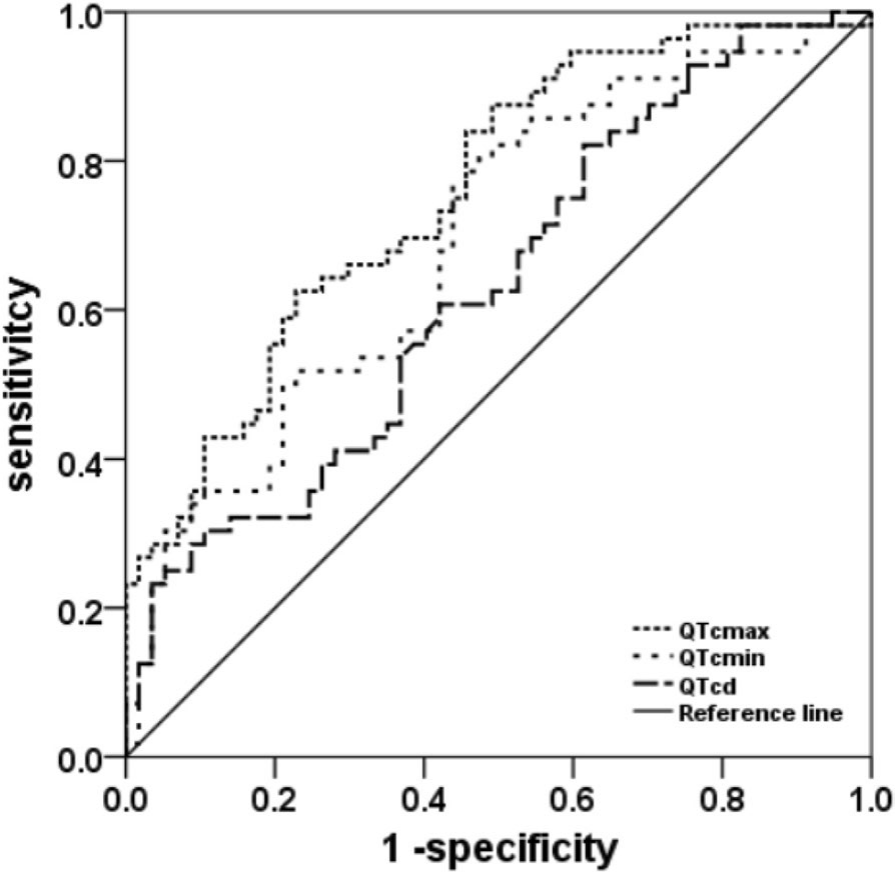
Efficacy of QT interval index in differential diagnosis of VVS and epilepsy in children. Note: QTcmax: corrected maximum QT interval. QTcmin: corrected minimum QT interval. QTcd: corrected QT interval dispersion

The ROC curve was drawn to evaluate the efficacy of QT interval index in the differential diagnosis of VVS and epilepsy in children. The results showed that QTcmax, QTcmin and QTcd had certain value in the differential diagnosis of VVS and epilepsy in children (*P<*0.05). When QTcmax > 479.84 ms, QTcmin > 398.90 ms, QTcd > 53.56 ms, the sensitivity and specificity of diagnosing VVS were 62.50% and 77.19%, 82.14% and 50.88%, 82.14% and 38.60%, respectively. The AUC of QTcmax judgment efficiency was higher than that of QTcd (*Z* = 2.352, *P<*0.05), but the AUC of QTcmin judgment power was not significantly higher than that of QTcd (*Z* = 1.859, *P>*0.05), suggesting that the discriminant efficiency of QTcmax was higher than that of QTcd and QTcmin, as shown in Table 1; Fig. 3.

**Table 1:** Efficacy of QT interval index in differential diagnosis of VVS and epilepsy in children

Index	AUC	*P*	95%*CI*	Optimal truncation value (ms)		Sensitivity (%)	Specificity (%)	Positive likelihood ratio	Negative likelihood ratio	Yoden index (%)
QTmax	0.512	0.829	0.404 ~ 0.619	403.00	30.36		80.70	1.57	0.86	11.06
QTmin	0.447	0.333	0.340 ~ 0.554	347.33	25.00		78.95	1.19	0.95	3.95
QTd	0.576	0.165	0.470 ~ 0.681	37.00	92.86		24.56	1.23	0.29	17.42
QTcmax	0.758	0.000	0.671 ~ 0.845	479.84	62.50		77.19	2.74	0.48	39.69
QTcmin	0.693	0.000	0.596 ~ 0.790	398.90	82.14		50.88	1.67	0.35	33.02
QTcd	0.631	0.016	0.529 ~ 0.733	53.56	82.14		38.60	1.34	0.46	20.74

Note: QTmax: maximum QT interval. QTmin: minimum QT interval. QTd: QT interval dispersion. QTcmax: corrected maximum QT interval. QTcmin: corrected minimum QT interval. QTcd: corrected QT interval dispersion. AUC: area under the curve. 95% *CI*: 95%confidence interval

## Discussion

TLOC is a common clinical emergency, which can be caused by a variety of causes, including syncope and non-syncope. The causes of syncope mainly include NMS, cardiogenic syncope and cerebrovascular diseases, while non-syncope causes mainly include psychogenic pseudosyncope and other diseases similar to syncope, as well as diseases with partial or complete loss of consciousness (such as epilepsy) [13]. Syncope is the main cause of TLOC in children, and VVS is the most common in children with syncope [14]. It can be seen that both VVS and epilepsy can be expressed as TLOC.

QT interval is the time from the beginning of QRS wave to the end of T wave, which is equivalent to the end of phase 0 to phase 3 of cardiomyocyte action potential. It is the total time of depolarization and repolarization of cardiomyocytes, reflecting the time of transmembrane action potential of ventricular muscle, mainly through the transport of ions across the membrane to form the action potential, and the corresponding waveform is formed by ECG on the body surface. Autonomic nervous system is closely related to cardiovascular system. At present, the main clinical detection methods of cardiovascular autonomic neuroregulatory function are HUTT and electrocardiographic indexes such as P wave dispersion (Pd), heart rate variability (HRV), ventricular late potential (VLP), QTd, T peak-T end interval (Tp-Te interval) and so on. QT interval is regulated by autonomic nerve, sympathetic nerve is excited, heart rate is increased, QT interval is shortened, but it is opposite when vagus nerve is excited. QTc is converted to heart rate-independent correction by Bazett’s formula, that is, corrected QT interval, which is an indicator of cardiac depolarization and repolarization, and QTc prolongation indicates cardiac repolarization delay. Sympathetic nerve activity can cause changes in the shape of T wave. When sympathetic nerve activity is enhanced, the peak of T wave moves backward, which increases QTd. Because QTd reflects the heterogeneity of repolarization or the instability of electrical activity of cardiomyocytes in different parts of the ventricle, when the recovery time of ventricular excitability is inconsistent, the difference of repolarization of cardiomyocytes increases, and QTd is prolonged, which is prone to conduction abnormalities and reentry, which leads to malignant arrhythmias or sudden cardiac death, which is of great significance in predicting the occurrence of malignant arrhythmias [12].

VVS is one of the common causes of NMS in children and adolescents. Abnormal regulation or dysfunction of autonomic nervous system is mainly caused by a variety of triggering factors, which leads to transient cerebral ischemia caused by vasodilation, TLOC and loss of body balance [5, 15]. The pathogenesis of VVS involves many aspects. Hypovolemia, autonomic nervous dysfunction, vasomotor dysfunction, Bezold-Jarisch reflex theory, endothelial dysfunction, neurohumoral factors, intestinal microflora and genetic factors are involved in the potential mechanism of VVS [16]. Dysfunction of autonomic nervous system regulation is the main cause of VVS. Current studies have shown that abnormal QT interval and its related indexes may occur in VVS. By comparing QTd and Pd between VVS and healthy children, Wang et al. [17] found that QTd and QTcd in children with VVS were 25.28% and 16.17% longer than those in healthy children, respectively. And malignant arrhythmias occurred in children with VVS. Xue et al. [18] found that the QTd and Pd of VVS patients in supine and orthostatic position were 38.3% and 35.42% longer than that of healthy individuals by comparing QTd and Pd in supine position, while Pd and QTd in upright position of VVS patients were 24.87% and 22.09% shorter than those in supine position, respectively. It shows that VVS patients are more prone to arrhythmias in supine position, and autonomic nervous function can affect QTd and Pd, especially when the posture changes. It indicates that this method can be used to evaluate the function of cardiac autonomic nerve. Zhang et al. [19] through the Logical regression model analysis of 176 patients (150 VVS patients and 26 cardiogenic pseudosyncope patients), the results showed that QTd, syncope duration and upright posture could distinguish VVS from psychogenic pseudosyncope, in which the area under the QTd curve was 0.892. Cardiogenic pseudosyncope usually had no autonomic nervous dysfunction, which further indicated that QTd could reflect cardiac autonomic nervous function. Sucu et al. [20] analyzed and compared the indexes of QTc, QTd and Tp-Te in 66 adult patients with neurocardiogenic syncope. It was showed that the indexes of QTc, QTd and Tp-Te in the HUTT positive group was longer than that in the HUTT negative group. In this study, QTcmax, QTcmin and QTcd in the VVS group were significantly longer than those in the control group, which was consistent with the above study. It can be seen that there is autonomic nervous dysfunction in children with VVS. This study showed that there was no significant difference in QTd between the two groups, which may be affected by heart rate.

Epilepsy is a chronic brain disease caused by repeated abnormal discharges of neurons in the brain. Long-standing uncontrolled epilepsy can lead to cardiac autonomic nervous dysfunction [21]. Chronic epilepsy is associated with permanent changes in cardiac electrophysiology, which may be an important cause of arrhythmia, tachycardia or bradycardia, or even cardiac arrest. During seizures and interictal periods, local and generalized seizures have been shown to lead to changes in autonomic nervous system activity [22, 23]. The structure of the central autonomic neural network, especially the insular process cortex of the amygdala, the gray and central nucleus around the aqueduct and various brainstem nuclei, is considered to be the basis of autonomic nervous dysfunction caused by seizures [24]. Abnormal brain discharges interfere with the transmission and transmission of cardiac electrical signals, and frequent epileptiform discharges in the brain can damage the cortical and subcortical structures that regulate the autonomic nervous system of the cardiovascular system [25, 26]. In addition, frequent seizures can also cause myocardial injury and increase the risk of myocardial fibrosis and cardiovascular autonomic nervous dysfunction. It can be seen that some children with frequent seizures can also have abnormal autonomic nervous function, resulting in abnormal ECG indexes. Li et al*.* [27] found that the abnormal rate of cardiovascular autonomic nervous function in patients with epilepsy during interictal period was 45.1%, and the higher the abnormal rate was with the longer the course of disease, and the indexes of HRV time domain analysis and nonlinear quantitative analysis in patients with abnormal cardiovascular autonomic nervous function were lower than those in the normal control group. At the same time, it was found that interictal QTd and QTcd in patients with epilepsy were longer than those in normal individuals [28]. Tosun et al*.* [29] detected 30 children with idiopathic epilepsy by A-ECG. It was found that the time domain measurement of HRV and the average HF value were significantly decreased, while the average LF and average LF/HF parameters was significantly increased. Dagar et al*.* [30] through the observation of interictal ECG in 103 patients with epilepsy, it was found that Pd, QTd, QTcd, Tp-Te dispersion (Tp-Ted) and Tp-Te/QTc ratio in patients with epilepsy were higher than those in healthy individuals. Neufeld et al. [31] studied 40 patients with long-term epilepsy and found that the QTc of patients with epilepsy was longer than that of healthy individuals. In contrast, a retrospective study of 195 patients admitted to the epilepsy surveillance department of Beth Israel Deaconness Medical Center in 2013 found that there was no significant difference in average QTc [32]. Yang et al*.* [33] through the simultaneous detection of 24-h ambulatory electroencephalogram (A-EEG) and A-ECG in children with epilepsy and syncope, it was found that 13 of 17 patients with epilepsy showed normal A-ECG and only 4 showed sinus tachycardia. Sevcencu et al*.* [34] also found that interictal arrhythmia in patients with chronic epilepsy is similar to that in healthy people. Similarly, this study also found that there was no significant difference in QTd and QTcd between the epilepsy group and the healthy people, suggesting that there was no obvious autonomic nervous dysfunction in patients with epilepsy, but at the same time, it was inconsistent with some current studies [28–30]. On the one hand, the selected children were all children with primary epilepsy, the course of disease was short, and there was no myocardial damage and structural damage of cardiovascular autonomic cortex. On the other hand, this part of the children did not have seizures during ECG examination and did not interfere with ECG activity. Therefore, whether there will be abnormal ECG indexes in children with epilepsy needs further study.

## Strengths and limitations

Although this study shows that the specificity of QTcd in distinguishing VVS from epilepsy in children is low, and its judgment efficiency is not particularly ideal, it is a non-invasive examination, and its results are quick and easy to obtain, so it has certain advantages. It can make a preliminary judgment for children with transient loss of sexual consciousness to a certain extent, and it is more suitable to be promoted in outpatient clinics and grass-roots hospitals. Therefore, the comprehensive use of QTcd, detailed clinical history, physical examination and other examination methods can help clinicians to distinguish VVS from epilepsy more quickly, but it cannot replace the clinical diagnosis and evaluation of medical workers. Due to the small sample size involved in this study, VVS and epilepsy cannot be classified into types and ages to find the best diagnostic cutoff values of QTd and QTcd. Therefore, in the future research, we should gradually expand the sample size, adopt the way of multi-center research, and constantly improve the inspection results.

## Conclusion

We found that QT interval related parameters (QTcmax, QTcmin, QTcd) are significantly prolonged in VVS group (*P* < 0.05). QTcmax, QTcmin and QTcd can assist in the differential diagnosis of VVS and epilepsy. When QTcmax > 479.84 ms, QTcmin > 398.90 ms, QTcd > 53.56 ms, the sensitivity and specificity of VVS are 62.50% and 77.19%, 82.14% and 50.88%, 82.14% and 38.60%, respectively. However, there is no significant difference in QTd, QTmax and QTmin between the two groups (*P* > 0.05), which may be affected by heart rate. Therefore, QT interval indicators have certain clinical value in differentiating VVS and epilepsy in children.

## Supplementary Information

**Additional file 1.**

## Data Availability

The datasets used and/or analyzed during the current study are available from the corresponding author on reasonable request.

## Abbreviations

VVS: Vasovagal syncope
TLOC: Transient loss of consciousness
NMS: Neurally mediated syncope
VVS-VI: Vasoinhibitory type vasovagal syncope
VVS-CI: Cardioinhibitory type vasovagal syncope
VVS-M: Mixed type vasovagal syncope
ECG: Electrocardiogram
HUTT: Head-up tilt test
EEG: Electroencephalogram
QTd: QT interval dispersion
QTmax: Maximum QT interval
QTmin: Minimum QT interval
QTc: Corrected QT interval
QTcd: Corrected QT interval dispersion
ROC: Receiver operating characteristic curve
