# Supplementary material for: The value of QT interval in differentiating vasovagal syncope from epilepsy in children
Source: Ital J Pediatr. 2022 Dec 12;48:197. doi: 10.1186/s13052-022-01388-2 (PMC9743691; doi:10.1186/s13052-022-01388-2)
Supplement: Supplementary file 1 — Additional file 1. [file 13052_2022_1388_MOESM1_ESM.doc]

**Cover Letter**

Dear editorial board of Ital J Pediatr,

We would like to submit the enclosed manuscript entitled “The value of QT interval in differentiating vasovagal syncope from epilepsy in children”, by Xin Wang, Shuo Wang, Haihui Xiao, Runmei Zou, Hong Cai, Liqun Liu, Fang Li, Yuwen Wang, Yi Xu, Cheng Wang, which we wish to considered for publication in Ital J Pediatr. We would like to declare that there are no prior publications or submissions with any overlapping information, including studies and patients. The manuscript has not been and will not be submitted to any other journal while it is under consideration by Ital J Pediatr. No potential conflict of interest exits in the submission of this manuscript.

No honorarium, grant, or other form of payment was given to anyone to produce the manuscript. Each author listed on the manuscript has seen and approved the submission of this version of the manuscript and takes full responsibility for the manuscript.

We deeply appreciate your consideration of our manuscript, and are looking forward to receiving comments from the reviewers. If you have any queries, please don’t hesitate to contact me at the address below.

Thank you and the best regards.

Yours sincerely,

Cheng Wang on behalf of the authors.

**Corresponding authors**: Prof. Cheng Wang
**E-mail**: wangcheng2nd@csu.edu.cn
**Tel:** 86 731 852 95258

**ORCID:** 0000-0002-7120-0654

Department of Pediatric Cardiovasology, Children's Medical Center, The Second Xiangya Hospital, Central South University, Changsha, Hunan, 410011, China.
